# Transferrin receptor 1 is a supplementary receptor that assists transmissible gastroenteritis virus entry into porcine intestinal epithelium

**DOI:** 10.1186/s12964-018-0283-5

**Published:** 2018-10-20

**Authors:** Shuai Zhang, Weiwei Hu, Lvfeng Yuan, Qian Yang

**Affiliations:** College of Veterinary Medicine, Nanjing Agricultural University, Wei gang 1, Nanjing, Jiangsu 210095 People’s Republic of China

**Keywords:** Transmissible gastroenteritis virus, IPEC-J2 cells, Transferrin receptor 1

## Abstract

**Background:**

Transmissible gastroenteritis virus (TGEV), the etiologic agent of transmissible gastroenteritis, infects swine of all ages causing vomiting and diarrhea, in newborn piglets the mortality rate is near 100%. Intestinal epithelial cells are the primary target cells of TGEV. Transferrin receptor 1 (TfR1), which is highly expressed in piglets with anemia, may play a role in TGEV infection. However, the underlying mechanism of TGEV invasion remains largely unknown.

**Results:**

Our study investigated the possibility that TfR1 can serve as a receptor for TGEV infection and enables the invasion and replication of TGEV. We observed that TGEV infection promoted TfR1 internalization, clustering, and co-localization with TfR1 early in infection, while TfR1 expression was significantly down-regulated as TGEV infection proceeded. TGEV infection and replication were inhibited by occluding TfR1 with antibodies or by decreasing TfR1 expression. TGEV infection increased in TGEV-susceptible ST or IPEC-J2 cell lines and TGEV-resistant Caco-2 cells when porcine TfR1 was over-expressed. Finally, we found that the TGEV S1 protein interacts with the extracellular region of TfR1, and that pre-incubating TGEV with a protein fragment containing the extracellular region of TfR1 blocked viral infection.

**Conclusions:**

Our results support the hypothesis that TfR1 is an additional receptor for TGEV and assists TGEV invasion and replication.

**Electronic supplementary material:**

The online version of this article (10.1186/s12964-018-0283-5) contains supplementary material, which is available to authorized users.

## Background

Transmissible Gastroenteritis Virus (TGEV), a porcine enteropathogenic coronavirus, can cause watery diarrhea, vomiting, and rapid dehydration. Infection with TGEV results in nearly 100% mortality in neonatal piglets [1, 2], and is responsible for enormous economic losses in the swine industry [3]. TGEV is an enveloped virus with a large positive-sense single-stranded RNA genome that encodes 4 structural proteins: spike protein (S), envelope protein (E), membrane protein (M), and nucleoprotein protein (N) [4, 5]. The structure of the S glycoprotein changes radically as it binds to specific epithelial cell receptors and mediates the fusion of cellular and viral membranes [5–8]. Significant effort has been made to characterize the cellular receptors for TGEV infection. Porcine aminopeptidase N (pAPN), also known as CD13, has been identified as a primary cell-surface receptor for TGEV [9–13]. Our laboratory recently reported that epidermal growth factor receptor (EGFR) also promotes clathrin-mediated endocytosis of TGEV [14]. TGEV may use a third protein (200 kDa), currently uncharacterized, for cell entry [15, 16]. Identification of this receptor would provide additional insight into TGEV-mediated disease and define a novel therapeutic target for protecting swine from TGEV infection.

TfR1 is a homo-dimeric type II transmembrane glycoprotein with two apparent 100 KDa subunits (total 200 KDa). TfR1 contains a large extracellular C-terminal domain (671 amino acids, includes a binding site for its ligand transferrin), a single transmembrane domain (28 amino acids), and a short intracellular N-terminal domain (61 amino acids) [17–19]. In normal intestinal epithelium, transferrin receptor 1 (TfR1/CD71) is predominantly expressed in the crypt enterocytes (observed on the basolateral surface and in the cytoplasm of crypt epithelial cells) [20–22], with little expression in the epithelial cells at the apical of the villus. In contrast, under conditions of iron-deficiency anemia and low iron stores, the iron-responsive element/iron regulatory protein (IRE/IRP) network prevents TfR1 mRNA degradation [23–27] and up-regulates TfR1 substantially, resulting in expression all across the intestinal epithelium [18, 28, 29]. In addition to its functions in iron uptake and homeostatic maintenance of the intestinal epithelium [18, 20, 30], TfR1 had also been identified as a cellular entry receptor for several viruses, including the New World arenaviruses, New World hemorrhagic fever arenaviruses, Machupo virus (MACV) [31, 32], mouse mammary tumor virus (MMTV) [33, 34], the canine and feline parvovirus, feline panleukopenia virus [35, 36], and hepatitis C Virus (HCV) [37]. Additionally, Junín virus (JUNV) uses TfR1 as a cellular receptor for entry into susceptible cells by clathrin-mediated endocytosis [38].

Because TfR1 is distributed widely along the surface epithelium of newborns with anemia, and intestinal epithelial cells of newborn piglets are targets of TGEV, it is possible that TfR1 is the as yet uncharacterized 200 kDa protein that mediates TGEV infection. To test this hypothesis, we used cultured porcine intestinal columnar epithelial cells (IPEC-J2), derived from the neonatal piglet mid-jejunum [39, 40], as a model to characterize the interaction between TfR1 and TGEV in vitro. Our results indicate that TfR1 serves as a receptor for TGEV, contributes specifically to virion binding and endocytosis, and determines TGEV tropism.

## Methods

### Antibodies and reagents

#### Antibodies

Rabbit anti-human TfR1 (Abcam). Mouse monoclonal antibodies to His HA, and GFP (CMCTAG, Milwaukee, USA). Rabbit monoclonal anti-TGEV N protein was prepared in our laboratory. Anti-GAPDH monoclonal antibody, HRP-conjugated goat anti-rabbit and anti-mouse IgG (H + L) (Vazyme, Nanjing, China). FITC-conjugated anti-TGEV polyclonal antiserum (VMRD). Anti-rabbit IgG antibody (Beyotime).

#### Reagents

Ferristatin II and LMP agarose (Sigma-Aldrich). Protease inhibitor cocktail, enhanced chemiluminescence (ECL), and Pierce BCA Protein Assay kit (Thermo Scientific). Protein A/G magnetic beads (B23201, Bimake, USA). Lenti-X HTX Packaging Mix (Clonetech). X-tremeGENE HP DNA Transfection Reagent (Roche, Switzerland). PVDF membrane (Millipore). NP-40 lysis buffer (Beyotime). TRIzol reagent (Takara, Dalian, China). RIPA lysis buffer and SDS-PAGE Sample Loading Buffer (5X) (FcMACS, NanJing, China).

### Cell culture

Caco-2 and IPEC-J2 cell lines (Guangzhou Jennio Biotech Co, Ltd., China), ST cell line (ATCC,USA), and HEK 293 T cell line (ATCC,USA) were cultured in Dulbecco’s Modified Eagle’s Medium (DMEM from Life Technologies) supplemented with 10% fetal bovine serum (FBS, GIBCO), 16 mM HEPES (Life Technologies), and 100 μg/ml penicillin/streptomycin (Invitrogen) in a humidified atmosphere containing 5% CO_2_ at 37 °C. Cells were routinely seeded at a density of 2 × 10^5^/mL in 25 cm^2^ plastic tissue culture flasks (Corning) and passaged every 3–4 days for a maximum of 30 passages.

### Virus strains

TGEV (strain SHXB) was provided by the Jiangsu Academy of Agricultural Sciences and propagated in ST cells. The complete TGEV SHXB genome sequence is available in GenBank (KP202848.1) [41]. Viruses were labeled with the fluorescent probe DyLight 488 NHS Ester (Thermo Scientific) according the manufacturer’s recommended protocol.

### Plasmid construction

A DNA fragment encoding TfR1 was amplified by PCR and then inserted into the pLVX-DsRed-Monomer-N1 expression vector (EcoRI/BamHI) (Clontech). Similarly, TfR1-Out (encoding the extracellular region of TfR1) was cloned into pAcGFP1-C (using SalI/BamHI) (Clontech) and pET-32a-c(+) (using EcoRI/SalI). TGEV Spike1 (S1) was cloned previously into pCMV-C-HA (BamHI/XbaI) in our laboratory [14]. ShRNA sequences targeted against TfR1 were designed using tools available at http://rnaidesigner.lifetechnologies.com/rnaiexpress/insert.do and BLOCK-iT RNAi Designer. The best silencing efficiency was exhibited by clone NM_214001.1 (*Sus scrofa* transferrin receptor 1). shRNAs were cloned into the pLVX-shRNA1 vector (EcoRI/BamHI) (Takara, Dalian, China). All primers used in PCR are described in Table 1.

**Table 1:** Primer sequences used for plasmids construction

Name	Primer sequence (5′-3′)	Vector
shTfR1	shTfR1:ggacatgctcatctaggaaca	pLVX-shRNA1
	shCtrl: gcttgcacttaaagtagtaga	
TfR1	F: ctcaagcttcgaattcatgatggatcaagctagat	pLVX-DsRed
	R: ggcgaccggtggatcccgttaaaattcattgtcaat	
TfR1-Out	F: tgatatcggatccgaattcgcctattgtaaacgtgtag	pET-32a-c(+)
	R: gcaagcttgtcgacaaattcattgtcaatgtc	
	F: cttaaggcctctgtcgacgcctattgtaaacgtgtag	pAcGFP1-C
	R: ccggtggatccgccagaattcttaaaattcattgtcaatgt	
TGEV-S1	F: tctagcccgggcggatcctgtgctagttatgtggct	pCMV-C-HA
	R: atcgtatgggtatctagaatttgtataattatatatagag	

### Virus infection

Cells were seeded with or without treatment for the indicated times, inoculated with TGEV at a MOI of 5 at 4 °C for 1 h, and then washed three times with cold phosphate-buffered saline (PBS) to remove unattached virus. The cells were shifted to 37 °C in a 5% CO_2_ incubator and maintained in DMEM supplemented with 2% FBS and 100 μg/ml penicillin/streptomycin. Infected cells and supernatants were harvested at the conclusion of the incubation period.

### Indirect immunofluorescence assay (IFA)

To determine whether TfR1 and TGEV co-localize, IPEC-J2 cells were seeded on cover slips in 24-well tissue culture plates, infected with TGEV or 488-conjugated TGEV at 4 °C for 1 h, and then incubated at 37 °C for various times. Cells were fixed in 4% paraformaldehyde for 10 min and incubated with 0.1% Triton X-100 in PBS for 5 min. After the cells were blocked with 5% bovine serum albumin (BSA), they were incubated with primary antibodies (1:1000) overnight at 4 °C. Cells were then rinsed and incubated with fluorochrome-conjugated secondary antibodies (1:200) for 30 min at room temperature, washed again three times with PBS and incubated with 1 μg/ml DAPI for 5 min. Images were captured using a Zeiss LSM710 confocal microscope (Carl Zeiss, Germany) and analyzed using ZEN 2012 (Blue edition) (Carl Zeiss).

### Western blotting

At the indicated times post infection, cells were washed with PBS and lysed in ice-cold cell lysis buffer with protease inhibitor cocktail. Total protein concentration was determined with a Pierce BCA Protein Assay kit, using the bicinchoninic acid spectrophotometric method. Samples containing equal amounts of protein were separated SDS-PAGE and transferred to a PVDF membrane. The membrane was blocked with 5% nonfat milk in Tris-buffered saline (TBS) containing 0.1% Tween 20, incubated overnight at 4 °C with primary antibodies (1:1000), and then incubated with the corresponding HRP-conjugated secondary antibodies (1:5000) for 60 min at 37 °C. Antibody binding was detected by autoradiography using ECL.

### Co-immunoprecipitation (co-IP) assay

IPEC-J2 cells were incubated in RIPA lysis buffer containing protease inhibitor cocktail and then centrifuged at 12,000×g for 15 min. The cleared lysate was pretreated with 2 μl anti-rabbit IgG control and fresh protein A/G magnetic beads for 1 h at 4 °C to eliminate nonspecific binding to the beads. The beads were removed by magnetic separator and the lysate was incubated with 1 μg of anti-TfR1 Ab overnight at 4 °C on a rocker platform. 20 μL of fresh protein A/G magnetic beads were then added to the mixture, and incubated for 2 h at 4 °C on a rocker platform. The beads were washed four times with PBS, combined with PAGE loading buffer, and incubated for 10 min at 100 °C to release the immunoprecipitated proteins. The proteins were analyzed by western blotting using anti-TfR1 Ab. In parallel, magnetic beads loaded with TfR1 were incubated with purified TGEV for 5 h at 4 °C on a rocker platform. Beads were washed four times with PBS, and prepared for western blotting using the same methods. The blot was probed with anti-TGEV-N Ab and anti-TfR1 Ab.

To identify the TfR1 binding domain, we co-transfected plasmids expressing TfR1 and TGEV-S1 (or TfR1-out and TGEV-S1) into HEK 293 T cells that had reached 80–90% confluence. Transfection was conducted using the X-tremeGENE HP DNA Transfection Reagent following the manufacturer’s instructions. After 24 h, cells were lysed in NP-40 lysis buffer containing a protease inhibitor cocktail then centrifuged at 12000×g for 10 min. The supernatant was pretreated with protein A/G magnetic beads and IgG (from the same species as the immunoprecipitating antibody) for 1 h at 4 °C to eliminate nonspecific binding to the beads. The beads were removed by centrifugation and the supernatant was then incubated on a rotary mixer overnight at 4 °C with the immunoprecipitating antibody. Fresh protein A/G magnetic beads were added to the mixture and incubation continued for 3 additional hours at 4 °C. The complexes were analyzed by western blotting using antibodies against HA, GFP, and TfR1.

### RNA isolation and RT-qPCR analysis

To examine virus entry, IPEC-J2 cells were infected with TGEV at a MOI of 5 for 1 h at 37 °C. TRIzol reagent was used to extract total RNA, following the manufacturer’s protocol. RNA was analyzed by RT-qPCR, as previously described [14]. Gene expression was calculated using the comparative Ct method and results from three independent experiments. RNA levels were normalized using endogenous GAPDH RNA as a reference. Primer sequences used for qRT-PCR are listed in Table 2.

**Table 2:** Primer sequences used for qRT-PCR

Name	Primer sequence (5′-3′)
TGEV-N	F: caattcccgtggtcggaaga
	R: tttacgttggcccttcacca
GAPDH	F: tcatcatctctgccccttct
	R: gtcatgagtccctccacgat

### Flow cytometry

Flow cytometry was used to detect TGEV entry into cells as follows: Fluorescently tagged TGEV was incubated with cells for 1 h at 4 °C. The cells were washed with PBS and maintained in DMEM for 1 h at 37 °C in 5% CO_2_, then harvested using 0.25% trypsin. Acquisition of the fluorescent cells done was by FACS (Becton Dickinson) and the data were analyzed using FlowJo software (Version 7.6.5).

### Gene transduction

To produce lentivirus, we transfected the TfR1 expression construct or shTfR1 into HEK 293 T cells using the Lenti-X HTX Packaging Mix (plasmids pLP1, pLP2, and VSV-G) and the X-tremeGENE HP DNA Transfection Reagent, following the manufacturer’s instructions. 12 h post transfection, the culture medium was renewed and incubation continued for 2 and 3 days. Virus-containing supernatants were collected, filtered (pore size 0.45 μm), and stored at − 70 °C.

IPEC-J2, ST, and Caco-2 cells were infected with lentiviral particles (MOI = 1) containing the TfR1 expression construct. Twenty-four hour post infection the culture medium was refreshed, and incubation continued for 12–24 h to allow for maximum protein expression.

IPEC-J2 and ST cells were transfected with the TfR1 shRNA (shTfR1) and the negative control (scrambled) vectors (shCtrl). To generate cells that were stably transformed with IPEC-J2-shTfR1 and ST-shTfR1, lentiviral particles (MOI of 1) were added to the cells in the presence of 8 μg/ml polybrene. After incubation for 8 h, the cell cultures were expanded and maintained for 2 weeks in DMEM with 5 μg/ml puromycin. Surviving cells were maintained in medium supplemented with 2 μg/ml puromycin. Lysates from transduced cells were analyzed by western blotting.

### Expression and purification of his-tagged TfR1-out recombinant protein

TfR1-Out recombinant protein was expressed in *Escherichia coli* BL-21 and purified using Ni-NTA resin, following the manufacturer’s protocol as previously described [14]. Expressed 32a protein was used as a control.

### Plaque assay

Confluent monolayers of ST cells in 12-well plates were inoculated with serial ten-fold dilutions of virus suspension and incubated for 1 h at 37 °C. The cells were then overlaid with 0.7% low melting point agarose in DMEM containing 2% FBS and incubated about 48 h at 37 °C. To visualize plaques, cells were stained with 1% crystal violet in methanol.

### Statistical analysis

Data are presented as means ± standard deviation (SD) from three independent experiments. Statistical analysis was performed using Statistical Program for Social Sciences (SPSS) 16.0. Differences between control and experimental groups were analyzed using Student’s *t*-test and one-way Analysis of Variance (ANOVA). Differences were considered statistically significant at * 0.01 < *p* < 0.05, ** *p* < 0.01.

## Results

### TfR1 co-localizes with TGEV and is expressed at lower levels during infection

**Fig. 1:**
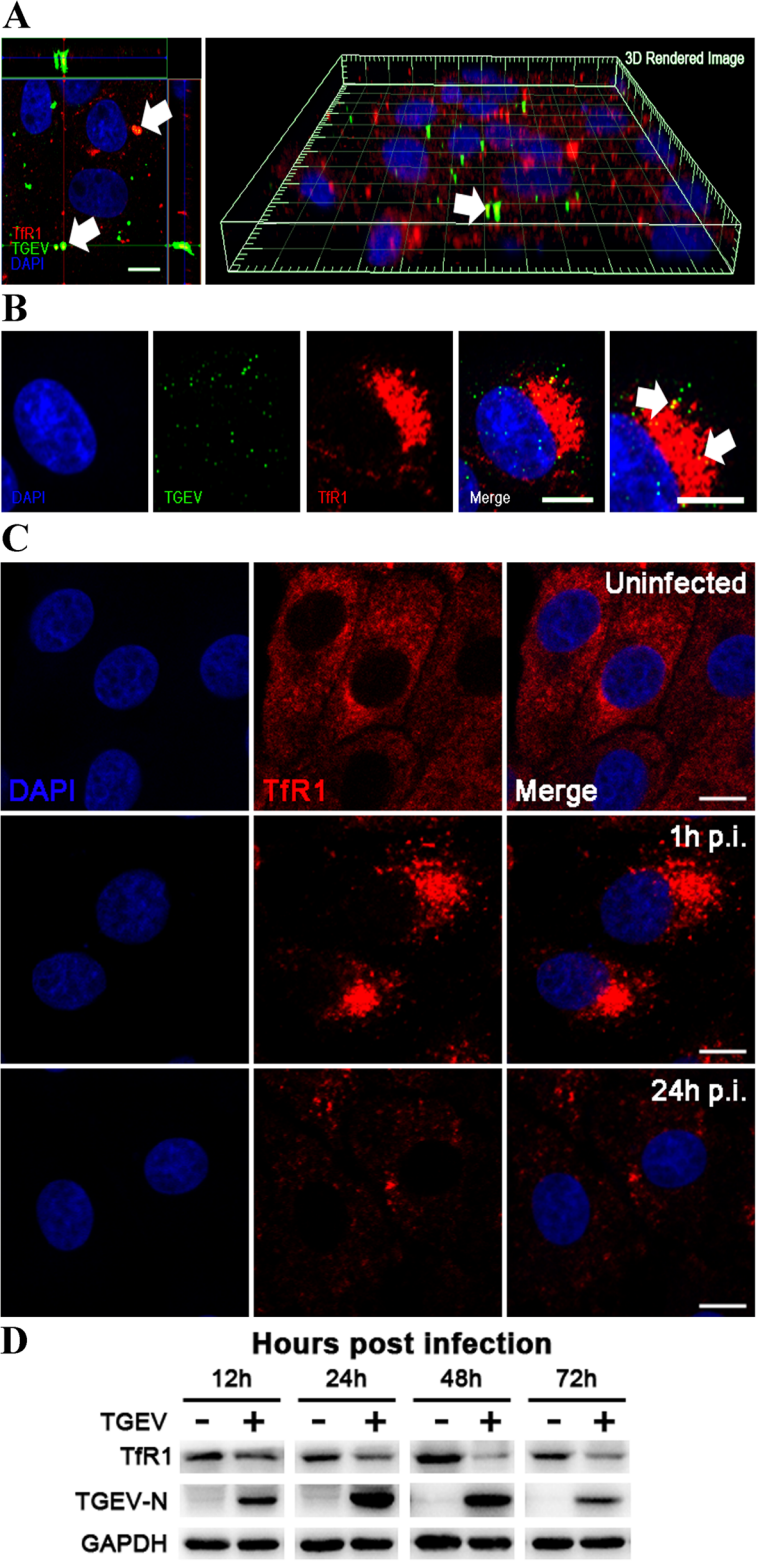
TGEV co-localizes with TfR1 and decreases its expression. **A** IPEC-J2 cells were infected with Dylight 488-TGEV (green), and cultured for 1 h. The cells were then stained for confocal microscopy using a rabbit anti-TfR1 pAb, followed by Dylight 633-conjugated goat anti-rabbit IgG (red). The panel shows a three-dimensional rendering of a representative field obtained using Imaris 7.2, and the arrows indicate co-localized signals (scale bar = 10 μm). **b** IPEC-J2 cells were infected with TGEV (MOI 5) and fixed at 1 h p.i. Cells were then stained for confocal microscopy using a rabbit anti-TfR1 Ab, followed by Dylight 633-conjugated goat anti-rabbit IgG (red) and FITC-conjugated anti-TGEV polyclonal antiserum (green). The arrows indicate co-localized signals (scale bar = 10 μm). **c** Indirect immunofluorescence images of TfR1 in mock- and TGEV-infected cells at 1 h p.i. and 24 h p.i. (MOI 5). Fixed cells were stained for TfR1 (red) and the nucleus was stained with DAPI (blue). Scale bar = 10 μm. **d** IPEC-J2 cells were uninfected or infected with TGEV (MOI 5) and harvested at different time intervals (12–72 h p.i.). The cell lysates were analyzed by western blotting using anti-TfR1, anti-TGEV-N, and anti-GAPDH antibodies as probes

To investigate whether TfR1 is involved in TGEV infection, we used confocal microscopy to visualize the subcellular locations of TGEV and TfR1 during TGEV infection. In the first experiment, TGEV was directly labeled with Dylight 488-labeled TGEV, while TfR1 was labeled indirectly using a Dylight 633-conjugated antibody (Fig. 1a). The results indicated that TfR1 co-localized with TGEV. The experiment was repeated using a dual-label immunofluorescence strategy (Fig. 1b). The results were consistent with our initial observation, and also showed that TfR1 appeared to re-localize, internalize, and cluster early in TGEV infection (Fig. 1b and c). Based on fluorescence, TfR1 protein levels were significantly reduced compared to levels in uninfected cultures at 24 h post infection (h p.i.) (Fig. 1c). Western blotting analysis confirmed that TfR1 expression decreased significantly until 72 h p.i. (Fig. 1d).

### Endogenous TfR1 interacts with TGEV

**Fig. 2:**
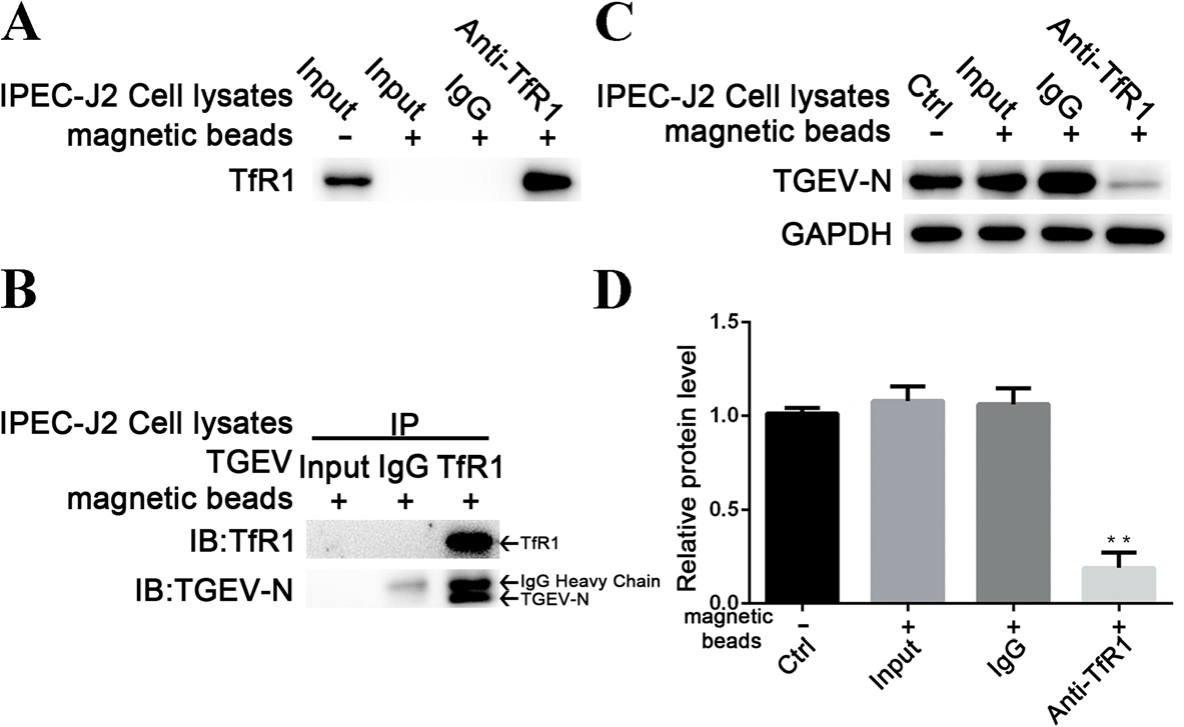
TGEV interacts with endogenous TfR1. **a** Endogenous TfR1 was enriched from IPEC-J2 cell lysates using magnetic beads coated with anti-TfR1 Ab. TfR1 immobilized on the beads was detected by western blotting. **b** The enriched endogenous TfR1 described in panel A was used as bait to bind TGEV. Empty magnetic beads and magnetic beads with allogeneic anti-rabbit IgG served as negative controls. The N protein of TGEV was precipitated using immobilized beads coated with anti-TfR1 Ab but not anti-rabbit IgG. **c** and **d** TGEV (MOI 5) was pre-incubated with the precipitated TfR1 protein for 2 h at 37 °C before cells were infected. At 24 h p.i., TGEV replication was analyzed by western blotting. Pre-incubation with TfR1 protein inhibited viral replication. The TGEV-N to GAPDH ratio was normalized to control conditions. Data shown are means ± SD from three independent experiments. (* 0.01 < *p* < 0.05, ** *p* < 0.01)

The co-localization of TfR1 and TGEV, and the decrease in TfR1 expression during TGEV infection, suggest that TfR1 and TGEV interact reciprocally. To test this hypothesis, endogenous TfR1 was enriched from IPEC-J2 cell lysates using magnetic beads bound to anti-TfR1 Ab. Empty magnetic beads (i.e., without antibody) and magnetic beads bound to allogeneic anti-rabbit IgG were used as negative controls (Fig. 2a). The beads were then tested for their ability to bind TGEV. Only beads that had previously captured endogenous TfR1 were able to bind TGEV (Fig. 2b). Importantly, pre-incubating TGEV (MOI 5) with the precipitated endogenous TfR1 blocked viral replication at 24 h p.i. (Fig. 2c and d). These data indicate that endogenous TfR1 interacts with TGEV.

### Blocking and suppressing TfR1 protein impairs infection by TGEV

**Fig. 3:**
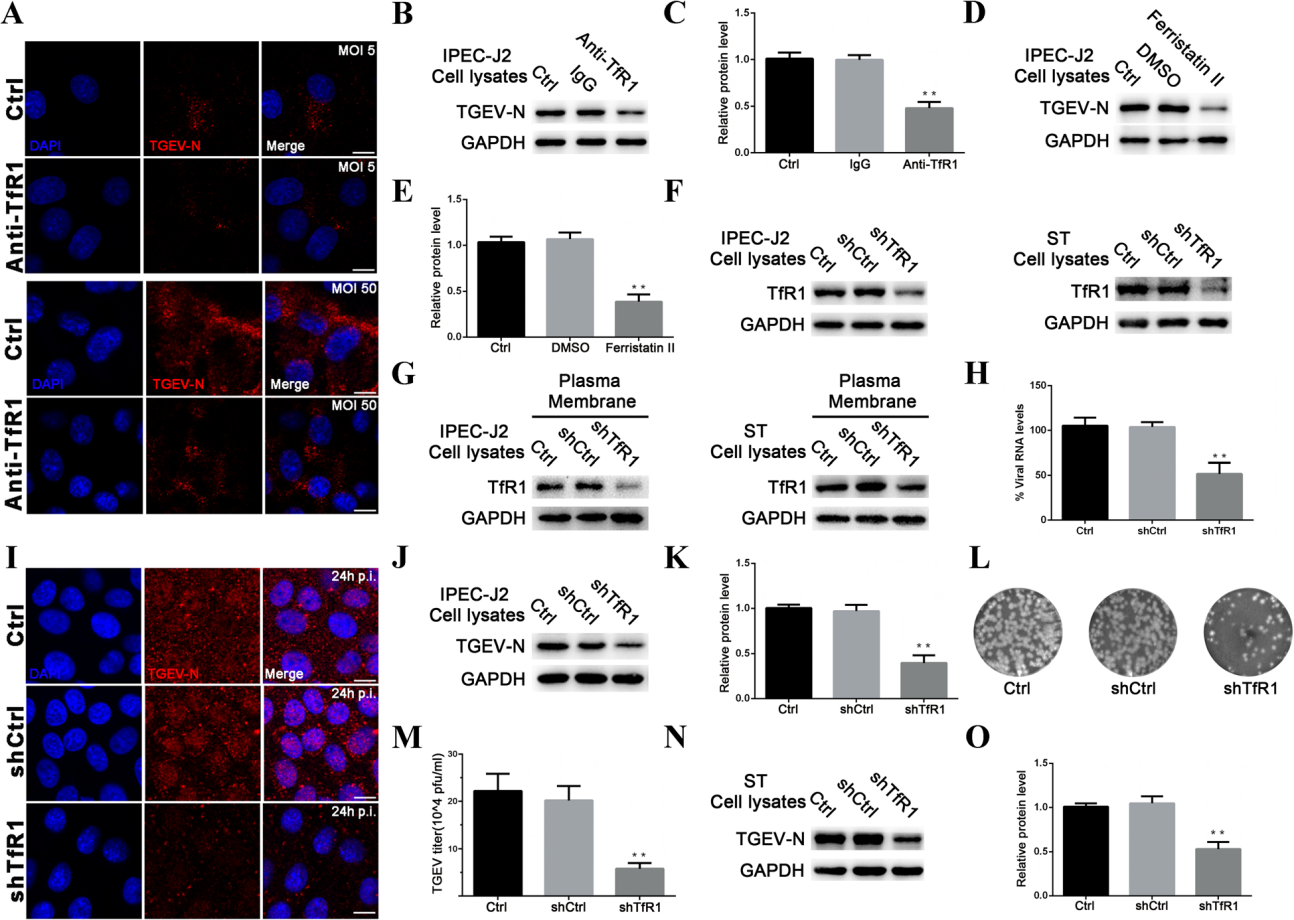
Blocking and Suppressing TfR1 protein inhibits TGEV Infection. **a** IPEC-J2 cells were pre-incubated with anti-TfR1 Ab for 1 h at 37 °C. Early invasion of TGEV (at 1 h p.i.; MOI 5 or 50) was inhibited. **b** and **c** IPEC-J2 cells were pre-incubated with anti-TfR1 Ab for 1 h at 37 °C. Replication of TGEV-N protein was detected at 24 h p.i. (MOI 5) by western blotting. **d** and **e** IPEC-J2 cells were pretreated with 50 μM ferristatin II and then infected with TGEV (MOI 5). For controls, cells that had not been pretreated with ferristatin II were infected with TGEV or pretreated with DMSO. Cell lysates were harvested for western blotting at 24 h. **f** and **g** Interference with total and membrane TfR1 levels in IPEC-J2 and ST cell lines were verified by western blotting. **h** IPEC-J2 cells were transfected with interference vector pLVX-shRNA-TfR1. pLVX-shRNA-Ctrl and normal cells served as controls. 1 h after infection with TGEV (MOI 5), TGEV-N mRNA levels were quantified by RT-PCR. **i** Confocal images of TGEV (MOI 5) infected IPEC-J2 cells transfected with shTfR1. Images were obtained 24 h p.i. The red stain indicates TGEV-N. Nuclei are stained with DAPI (blue). Scale bar = 10 μm. **j** and **k** shRNA was used target TfR1 in IPEC-J2 cells. The cells were infected with TGEV (MOI 5), and analyzed at 24 h p.i. by western blotting. **l** and **m** Culture supernatants from Fig **j** were harvested and virus titers were measured by plaque assay in ST cells. Plaques developed at 2 days after infection. Untreated cells and cells transfected with scrambled shRNA cells served as controls. **n** and **o** ST cells stably carrying the shTfR1 interference vector were infected with TGEV (MOI 5) and harvested at 24 h p.i. for western blotting. TGEV replication was inhibited by TfR1 knockdown. The TGEV-N to GAPDH ratio was normalized to control conditions. The data shown are means ± SD from three independent experiments. (* 0.01 < *p* < 0.05, ** *p* < 0.01)

To verify that TfR1 is involved in TGEV infection, we assessed the ability of TGEV (MOI 5 or 50) to infect cells after blocking cellular TfR1 with anti-TfR1 Ab. The cells were fixed, stained for TGEV-N protein using a fluorescence-tagged antibody, and then examined by microscopy. Fluorescence was significantly decreased at 1 h p.i. in blocked cells (Fig. 3a). At 24 h p.i., western blotting showed that expression of TGEV-N protein was significantly lower in IPEC-J2 cells that had been pre-incubated with anti-TfR1 Ab (Fig. 3b and c). In contrast, TGEV invasion was not affected by treatment with the TfR1 ligands holo-Tf or apo-Tf (Additional file 1: Figure S1).

Ferristatin II, a TfR1 inhibitor that causes degradation of TfR1 (34), was used to confirm the role of TfR1 in TGEV invasion. Preliminary experiments showed that a 50 μM dose of ferristatin II did not result in detectable cytotoxicity (Additional file 1: Figure S2). TGEV replication was significantly inhibited in IPEC-J2 cells pre-incubated with this concentration of ferristatin II at 24 h p.i. (Fig. 3d and e).

Given the evidence that TfR1 functions in TGEV infection, we performed analogous experiments using shRNA to target TfR1 in IPEC-J2 and ST cell lines. Both total TfR1 protein (Fig. 3f) and membrane TfR1 protein (Fig. 3g) decreased in the knockdown cells compared to normal cells. TGEV-N mRNA levels were also significantly lower in TfR1 knockdown cells at 1 h p.i. (Fig. 3h). To determine whether TfR1 knockdown affects TGEV replication in IPEC-J2 cells, we used immunofluorescence staining to detect changes in TGEV-N protein levels. TGEV-N protein was obviously reduced in cells treated with the TfR1 knockdown shRNA at 24 h p.i., relative to cells treated with the scrambled shRNA control (Fig. 3i). Likewise, we observed a significant decrease in TGEV replication in western blotting assays (Fig. 3j and k) and plaque assays (Fig. 3l and m) at 24 h p.i. Similar results were also obtained in ST cells (Fig. 3n and o).

### Overexpression of TfR1 enhances TGEV infection

**Fig. 4:**
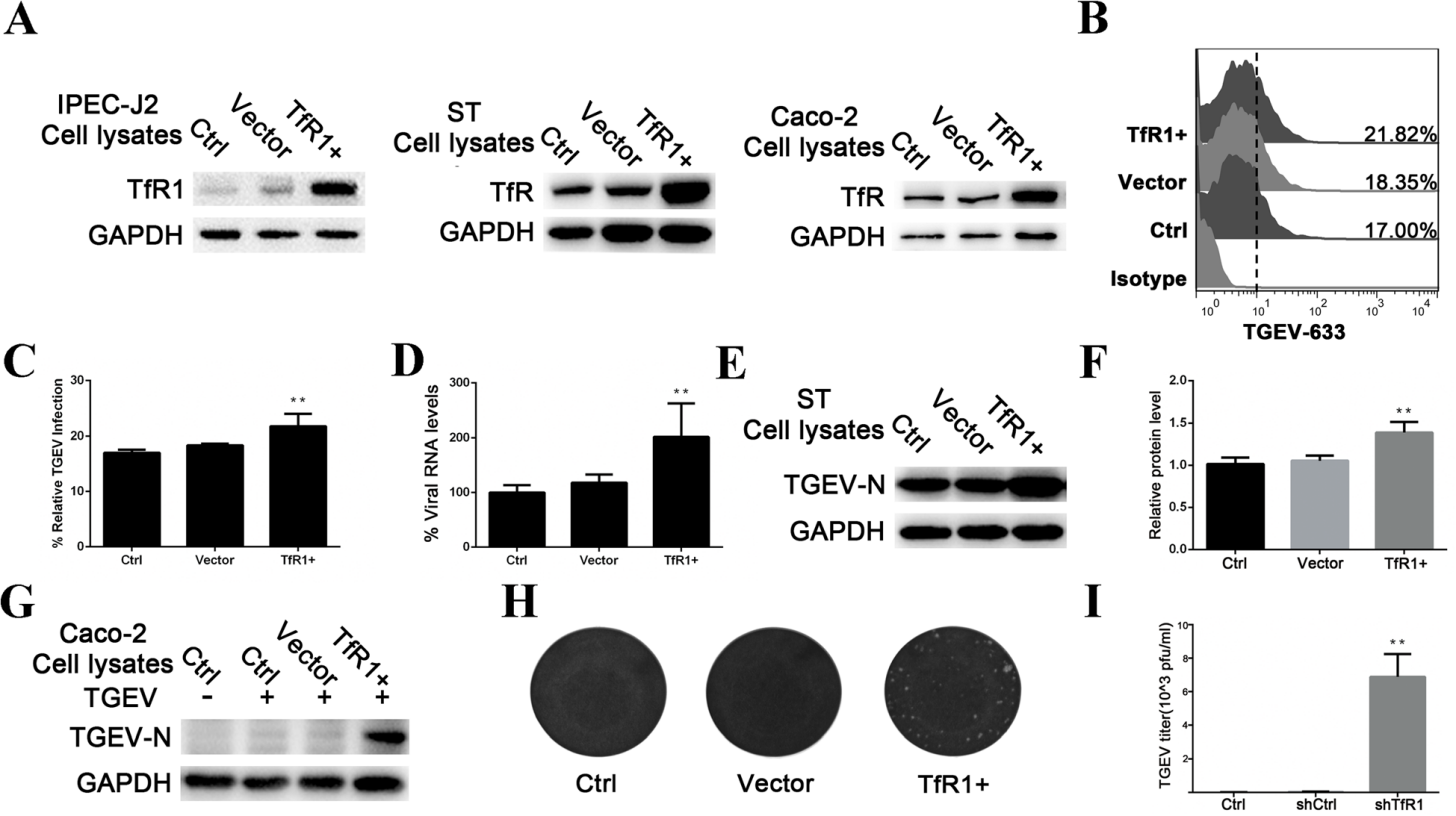
Overexpression of TfR1 enhances TGEV infection. **a** IPEC-J2, ST, and Caco-2 cells were transiently transfected with porcine TfR1. Cell lysates were analyzed for expression of TfR1 by western blotting using anti-TfR1 and GAPDH antibodies as probes. **b** and **c** IPEC-J2 cells that were overexpressing TfR1 were infected with Dylight 488-TGEV. After 1 h, TGEV invasion was detected by flow cytometry. **d** TfR1-overexpressing IPEC-J2 cells were infected with TGEV (MOI 5). After 1 h, TGEV-N mRNA levels were measured by RT-qPCR. **e** and **f** ST cells in which TfR1 was overexpressed were infected with TGEV for 24 h and lysates harvested for western blotting. Normal cells and cells transfected with expression vector alone served as controls. The TGEV-N to GAPDH ratio was normalized to control conditions. **g**-**i** TGEV-resistant Caco-2 cells that were expressing porcine TfR1 protein were challenged with TGEV (MOI 5). 24 h after infection, cell lysates were harvested for western blotting and culture supernatants were collected for viral plaque assays on ST cells. Plaques developed 2 days after infection. Normal cells and cells transfected with expression vector alone served as controls. The data shown are means ± SD from three independent experiments. (* 0.01 < *p* < 0.05, ** *p* < 0.01)

Because the experiments described above indicate that TfR1 is involved in TGEV infection, we investigated whether overexpression of TfR1 would enhance TGEV infectivity. IPEC-J2 and ST cells were infected with lentiviruses (MOI 1) containing the TfR1 expression construct or the empty control vector. As expected, cells infected with the TfR-expressing lentivirus particles showed significantly increased TfR1 expression over cells infected with lentivirus containing the empty vector (Fig. 4a).

To determine whether overexpression of TfR1 impacts the TGEV infection process, we used flow cytometry to test the ability of Dylight 488-labeled TGEV to associate with IPEC-J2 cells (Fig. 4b and c). TGEV mRNA levels were also were measured in IPEC-J2 cells at 1 h p.i. using RT-qPCR (Fig. 4d). Both experiments demonstrated that overexpression of TfR1 protein significantly increases viral infectivity. TGEV replication was also significantly higher in TfR1-overexpressing ST cells at 24 h p.i. (Fig. 4e and f). Finally, when TfR1 was overexpressed in TGEV-resistant Caco-2 cells, they became susceptible to TGEV infection (Fig. 4g, h and i).

### Extracellular TfR1 interacts with TGEV S1 protein in vitro

**Fig. 5:**
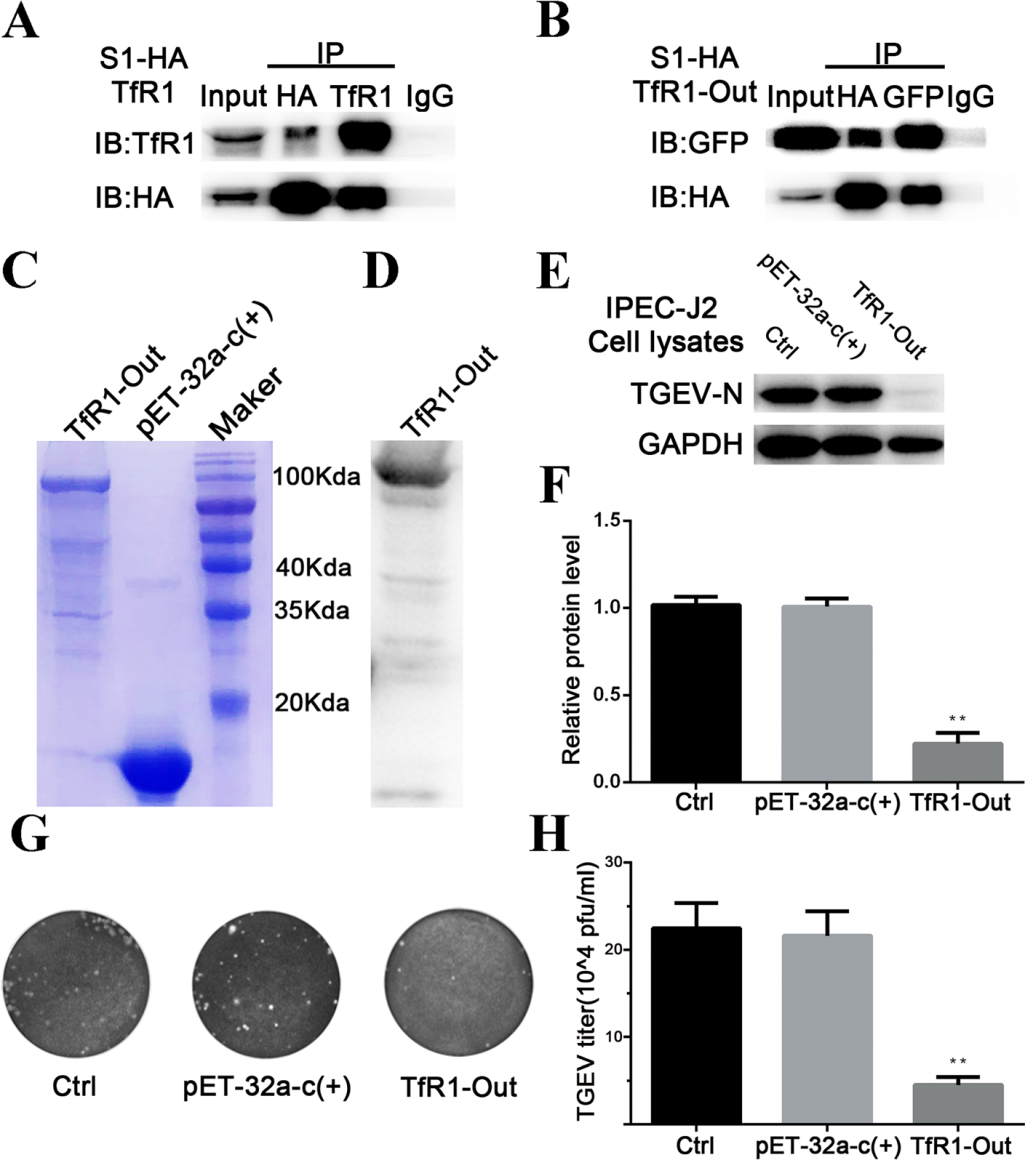
Extracellular TfR1 interacts with TGEV S1 protein in vitro. **a** and **b** 293 T cells were co-transfected with a HA-tagged TGEV S1 expression plasmid together with plasmids expressing TfR1 or GFP-tagged TfR1-Out. Cell lysates were immunoprecipitated with anti-HA antibody and anti-TfR1 antibody, or anti-HA antibody and anti-GFP antibody, respectively. The precipitates were examined by western blotting using anti-HA antibody and anti-TfR1 antibody (**a**) or anti-HA antibody and anti-GFP antibody (**b**) to examine the interaction between HA-TGEV-S1 and TfR1, or HA-TGEV-S1 and GFP-tagged TfR1-Out, respectively. **c** His-tagged TfR1-Out was expressed in E.coli BL21 and purified using a Ni-NTA column. Purified products were separated using SDS-PAGE and stained with Coomassie brilliant blue. **d** Purified TfR1-Out was verified by western blotting. **e** and **f** IPEC-J2 cells infected with TGEV (MOI 5) were pre-incubated with TfR1-Out (200 ng/mL) for 1 h at 37 °C, and cell lysates were harvested for western blotting. The ratio of TGEV-N to GAPDH was normalized to control conditions, and culture supernatants were collected for viral plaque assays in ST cells. Plaques developed 2 days after infection. Normal cells and cells treated with pET-32a-c(+) served as controls. Data shown are means ± SD from three independent experiments. (* 0.01 < *p* < 0.05, ** *p* < 0.01)

TGEV S1 protein binds to specific receptors on the membrane of susceptible cells [8]. To examine the interaction between TGEV S1 and TfR1, we obtained lysates from 293 T cells that had been co-transfected with plasmids expressing TGEV S1-HA and TfR1, and then conducted a co-immunoprecipitation assay. The results confirmed that TGEV S1 directly interacts with TfR1 (Fig. 5a). We then constructed a plasmid encoding the extracellular region of TfR1 (designated TfR1-Out) and transfected it into cells along with the TGEV S1 plasmid described earlier. The co-precipitation assay confirmed that the extracellular region of TfR1 interacts with TGEV S1 protein in vitro (Fig. 5b).

Finally, His-tagged TfR1-Out fusion protein was prepared using an *E. coli* expression system. Protein quality was verified by SDS-PAGE (Fig. 5c) and western blotting (Fig. 5d). When cells were pre-incubated for 2 h with TfR1-Out (200 ng/mL) prior to infection by TGEV, viral replication as reflected by TGEV-N levels, was inhibited (Fig. 5e and f). This result was consistent with a plaque assay for virus particles present in the cell culture medium (Fig. 5g and h).

## Discussion

TGEV invades the epithelial cells of the intestine via a receptor-mediated fusion mechanism [2, 6]. The species-specific virus tropism or host-range is usually determined by entry receptors [42]. The intestinal epithelium of neonatal piglets is particularly susceptible to TGEV [43]. Identifying the proteins that mediate the association between the host cell and the virus is therefore a crucial step for understanding virus-host interactions. In this study, we conducted experiments to determine if TfR1 can function as a receptor for TGEV invasion. The results show that TGEV induces the internalization, clustering, and down-regulation of cellular TfR1. Overexpression of TfR1 enhances TGEV invasion, and infection by TGEV can be inhibited if access to TfR1 is blocked, or if TfR1 levels are reduced. Finally, we determined that TGEV-S1 protein interacts with the extracellular region of TfR1. Together, the results support the conclusion that TGEV utilizes TfR1 to infect target cells.

Recently, Li et al. observed that the ability of TGEV to bind MDCK cells is enhanced when the cells express porcine aminopeptidase N (pAPN) [44]. We found that overexpression of TfR1 in the refractory Caco-2 cell line is sufficient to allow TGEV entry, synthesis of viral RNA and protein, and release of infectious TGEV. pAPN has been shown to function as a receptor for TGEV infection [9–12]. However, this protein seems to be widely distributed on enterocytes and probably on other tissues, irrespective of age [15]. An entry receptor or co-receptor usually mediates virus entry, as is the case for human immunodeficiency virus (HIV) and poliovirus [45–48]. A 200 kDa protein that is restricted to the villous enterocytes of newborn pigs has been identified as a possible additional receptor for TGEV, and may account for the age sensitivity of these animals to the virus [15]. Newborn piglets with iron-deficiency anemia usually present elevated levels of TfR1 on the entire surface of the intestinal epithelium [18, 28, 29]. TfR1 may therefore be a major contributing factor to the high level of TGEV susceptibility exhibited by anemic newborn pigs.

TfR1 plays a role in HCV entry via the clathrin endocytic pathway, and the expression of TfR1 is down-regulated in HCV-infected Huh7 cells [37]. JUNV also uses TfR1 as a cellular receptor for entry into Vero cells and is rapidly internalized by clathrin-mediated endocytosis [49], and canine parvovirus (CPV) utilizes limited TfR1 clustering on the surface to enter host cells through the clathrin endocytic pathway [36, 50]. Studies in our laboratories have demonstrated that EGFR is another promoter for TGEV entry via the clathrin-dependent endocytic pathway [14]. Upon binding to its ligand holo-Tf (iron-bound transferrin), TfR1 that is associated with clathrin-coated pits of the plasma membrane is internalized by dynamin-mediated endocytosis. The small GTPase Rab12, and its upstream activator Dennd 3, regulate membrane trafficking of TfR from recycling endosomes to lysosomes [51]. We observed that a decrease in TfR1 expression follows TGEV invasion. We hypothesize that the decrease occurs because TfR1 is degraded by lysosomes after binding to TGEV, and that TfR1 plays a critical role in the clathrin-mediated endocytosis of TGEV.

One of the four structural proteins encoded by the TGEV genome is the large transmembrane S glycoprotein. Trimers of S molecules form the surface spikes on coronaviruses which mediate virus entry and is a primary determinant tissue tropism cells [7, 52]. The ectodomain of the S protein consists of an N-terminal variable domain called S1 that is responsible for receptor binding, and a C-terminal conserved domain called S2 that is responsible for fusion. [8, 44, 53]. The TGEV-S1 protein interacts with pAPN and EGFR [53, 54]. In our study, we demonstrated that TGEV interacts with endogenous TfR1 and that TGEV-S1 protein interacts with TfR1 and the extracellular region of TfR1. The extracellular region of TfR1 is located between amino acid residues 90–760. Our experiments confirmed that pre-incubating TGEV with endogenous TfR1 or soluble TfR1-Out blocked viral invasion, supporting the hypothesis that these extracellular amino acids residues are important for the recognition of TGEV S1. In addition, the anti-TfR1 antibody used in our blocking experiments recognizes amino acid residues 1–100 of TfR1. This reagent also blocks TGEV invasion, presumably by occluding the site at which TGEV binds to TfR1. In contrast, neither holo-Tf nor apo-Tf interfered with the ability of TGEV to infect IPEC-J2 cells, indicating the use of non-overlapping binding sites. Based on these results, we hypothesize that TfR1 amino acid residues 90–100 encode the binding site for S1 protein.

## Conclusions

Our experimental results strongly support the conclusion that TfR1 functions as a receptor for TGEV invasion. The results are also consistent with the hypothesis that TfR1 may be responsible for the high level of TGEV susceptibility exhibited by newborn pigs. Additional work will be required to verify this hypothesis. Finally, this study will facilitate the development of vaccines to combat TGEV infection.

## Additional file

Additional file 1: **Figure S1.** TGEV invasion didn't affect with the treatment of TfR1 ligands, holo-Tf and apo-Tf. **Figure S2.** The cytotoxic test of IPEC-J2 cells and the inhibitory effect of Ferristatin II. (DOCX 1138 kb)

## Abbreviations

EGFR: Epidermal growth factor receptor
IPEC-J2: Porcine intestinal columnar epithelial cells
pAPN: Porcine aminopeptidase N
TfR1: Transferrin receptor 1
TGEV: Transmissible gastroenteritis virus
