# Supplementary material for: Transferrin receptor 1 is a supplementary receptor that assists transmissible gastroenteritis virus entry into porcine intestinal epithelium
Source: Cell Commun Signal. 2018 Oct 20;16:69. doi: 10.1186/s12964-018-0283-5 (PMC6196004; doi:10.1186/s12964-018-0283-5)
Supplement: Supplementary file 1 — Figure S1. TGEV invasion didn't affect with the treatment of TfR1 ligands, holo-Tf and apo-Tf. Figure S2. The cytotoxic test of IPEC-J2 cells and the inhibitory effect of Ferristatin II. (DOCX 1138 kb) [file 12964_2018_283_MOESM1_ESM.docx]

**
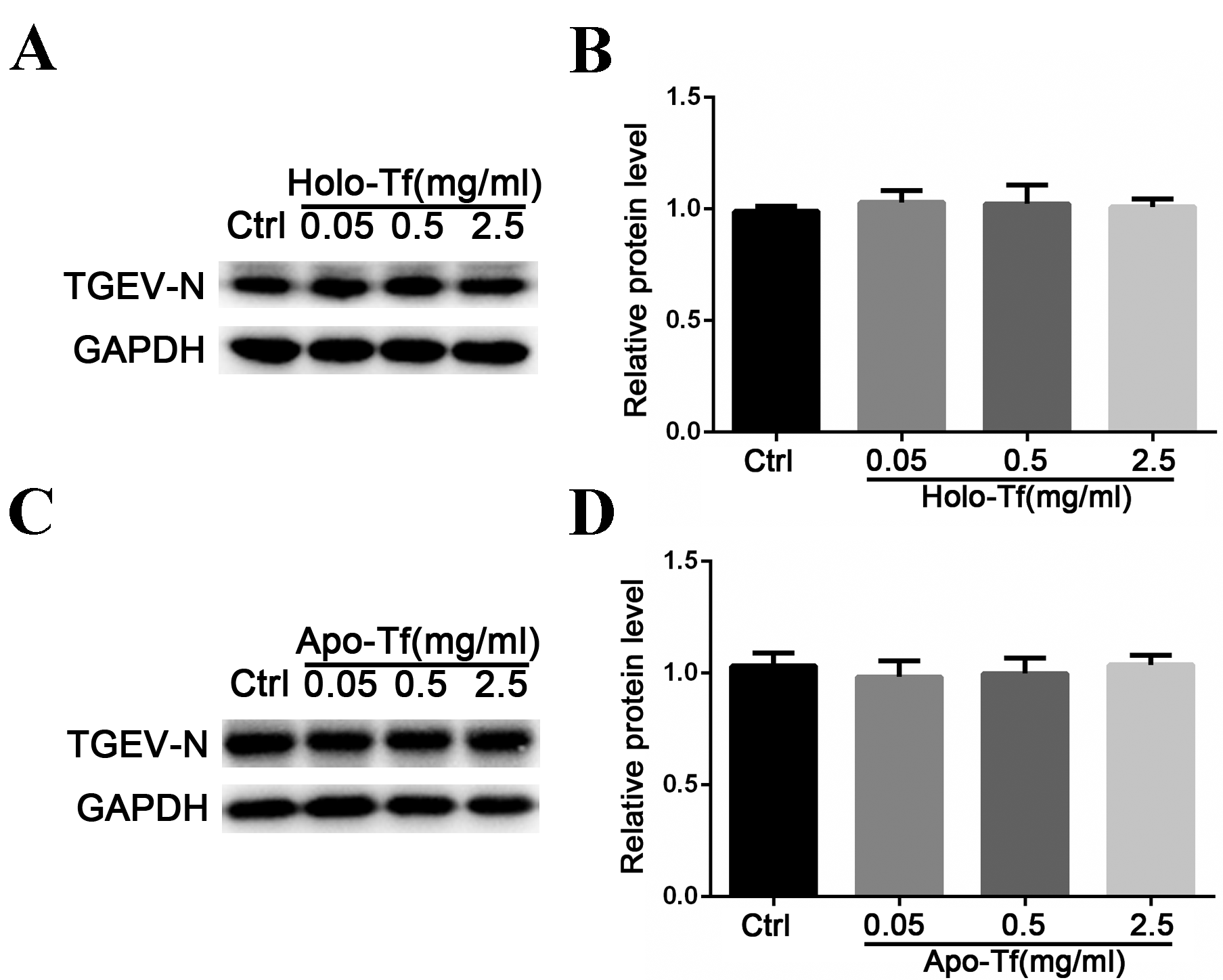
**

**Figure S1.** IPEC-J2 cells were pre-incubated with 0.05-2.5 mg/ml holo-Tf (A and B) or 0.05-2.5mg/ml apo-Tf (C and D) for 1 hour at 37 °C. The cells were washed and then infected with TGEV (MOI 5). TGEV replication was assessed at 24 h p.i. by western blotting. The ratio of TGEV-N to GAPDH was normalized to control conditions. Data shown are the means ± SD from three independent experiments. (* 0.01 < p < 0.05, ** p < 0.01).


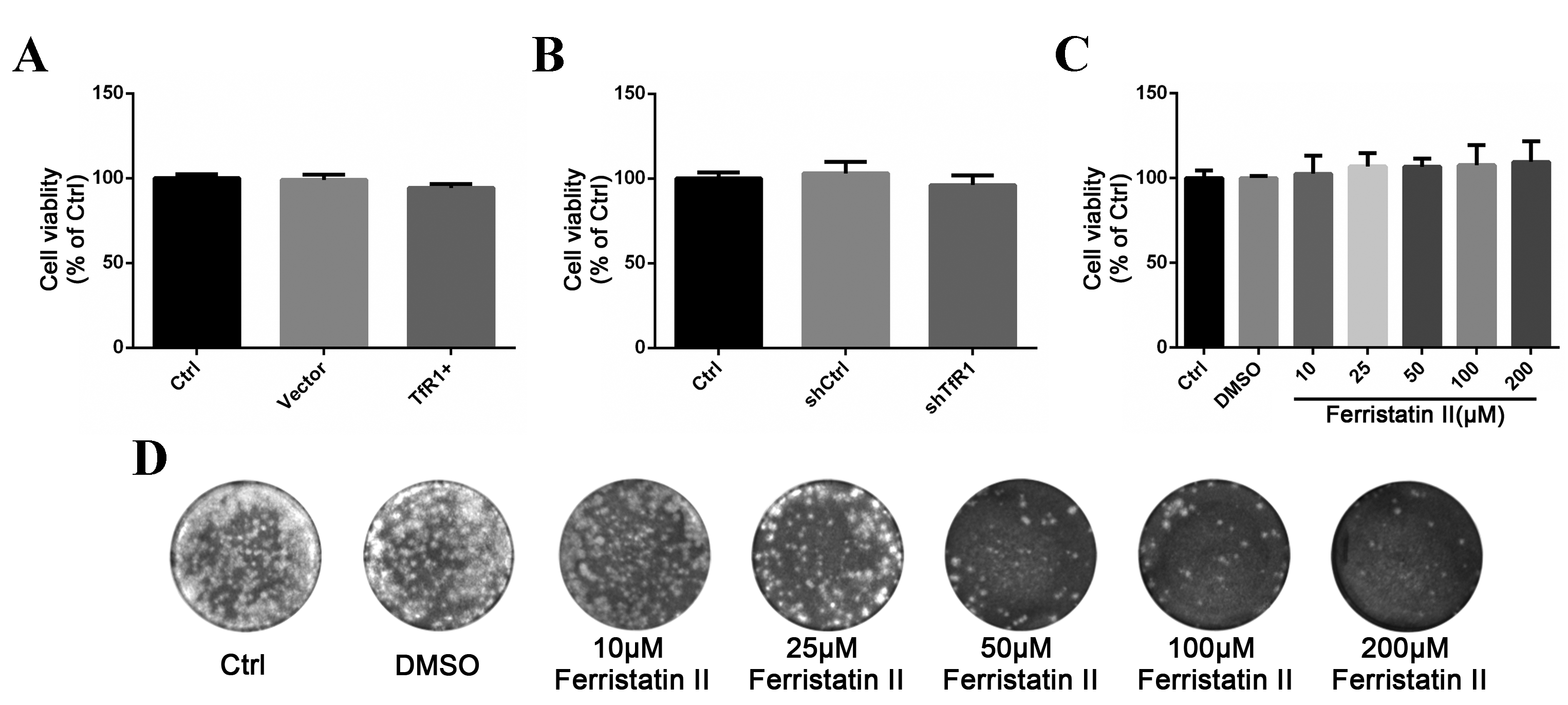


**Figure S2.** (A) IPEC-J2 cells were seeded in 96-well plates and treated with TfR1-overexpressing lentiviral particles (MOI 1). After 24 h, infected cells were maintained in fresh medium and incubated 12-24 h to achieve maximum effects. Cell viability was determined by CCK8 assay. Normal cells and cells treated with vector-only lentiviral particles (MOI 1) served as controls. (B) Stably transduced IPEC-J2-shTfR1 cells were seeded in 96-well plates. Cell viability was determined by CCK8 assay. Normal cells and the transduced IPEC-J2-shCtrl cells served as controls. (C) IPEC-J2 cells were seeded in 96-well plates. After incubation with ferristatin II (10-200 µM) for 1 h, cell viability was determined by CCK8 assay. (D) IPEC-J2 cells were pre-incubated with ferristatin II (10–200 µM) for 1 h at 37 °C, washed, and infected with TGEV (MOI 5) for 24 hours. Culture supernatants were collected and viral titers were determined by viral plaque assays in ST cells. Plaques developed 2 days after infection. Normal cells and cells treated with DMSO served as controls.
